# Human Papillomavirus and Oropharyngeal Cancer Among Indigenous Australians: Protocol for a Prevalence Study of Oral-Related Human Papillomavirus and Cost-Effectiveness of Prevention

**DOI:** 10.2196/10503

**Published:** 2018-06-08

**Authors:** Lisa Jamieson, Gail Garvey, Joanne Hedges, Amanda Mitchell, Terry Dunbar, Cathy Leane, Isaac Hill, Kate Warren, Alex Brown, Xiangqun Ju, David Roder, Richard Logan, Newell Johnson, Megan Smith, Annika Antonsson, Karen Canfell

**Affiliations:** ^1^ Australian Research Centre for Population Oral Health Adelaide Dental School University of Adelaide Adelaide Australia; ^2^ Menzies School of Health Research Charles Darwin University Darwin Australia; ^3^ Aboriginal Health Council of South Australia Adelaide Australia; ^4^ Yaitya Purruna Indigenous Health Unit University of Adelaide Adelaide Australia; ^5^ Aboriginal Health Division Women's and Children's Health Network Adelaide Australia; ^6^ Pika Wiya Health Service Inc Port Augusta Australia; ^7^ Wardliparingga Aboriginal Research Unit South Australian Health & Medical Research Institute Adelaide Australia; ^8^ School of Health Sciences Univesity of South Australia Adelaide Australia; ^9^ Adelaide Dental School University of Adelaide Adelaide Australia; ^10^ Menzies Health Institute Griffith University Gold Coast Australia; ^11^ Cancer Council of New South Wales Sydney Australia; ^12^ QIMR Berghofer Medical Research Institute QIMR Berghofer Medical Research Institute Brisbane Australia

**Keywords:** Papillomaviridae, oropharyngeal neoplasms, vaccination, population

## Abstract

**Background:**

Oropharyngeal cancer is an important, understudied cancer affecting Aboriginal and Torres Strait Islander Australians. The human papillomavirus (HPV) is a significant risk factor for oropharyngeal cancer. Current generation HPV vaccines are effective against the 2 most common types of high-risk HPVs in cancer (hrHPVs 16/18).

**Objectives:**

This study aims (1) to yield population estimates of oncogenic genotypes of HPV in the mouth and oropharynx of defined Aboriginal and Torres Strait Islander populations; (2) to estimate the proportion of oropharyngeal cancer attributable to HPV among these Australian citizens; (3) to estimate the impact of HPV vaccination as currently implemented on rates of oropharyngeal cancer among Aboriginal and Torres Strait Islander Australians; and (4) taking into account impact on oropharyngeal as well as cervical cancer, to evaluate efficacy and cost-effectiveness of targeted extended HPV vaccination to older ages, among our study population.

**Methods:**

Our study design and operation is straightforward, with minimal impost on participants. It involves testing for carriage of hrHPV in the mouth and oropharynx among 1000 Aboriginal South Australians by simple saliva collection and with follow-up at 12 and 24 months, collection of sexual history at baseline, collection of information for estimating health state (quality-of-life) utilities at baseline, genotyping of viruses, predictive outcome and cost-effectiveness modeling, data interpretation and development of vaccination, and follow-up management strategies driven by the Aboriginal community.

**Results:**

Participant recruitment for this study commenced in February 2018 and enrollment is ongoing. The first results are expected to be submitted for publication in 2019.

**Conclusions:**

The project will have a number of important outcomes. Synthesis of evidence will enable generation of estimates of the burden of oropharyngeal cancer among Aboriginal and Torres Strait Islander Australians and indicate the likely effectiveness and cost-effectiveness of prevention. This will be important for health services planning, and for Aboriginal health worker and patient education. The results will also point to important areas where research efforts should be focused to improve outcomes in Aboriginal and Torres Strait Islander Australians with oropharyngeal cancer. There will be a strong focus on community engagement and accounting for the preferences of individuals and the community in control of HPV-related cancers. The project has international relevance in that it will be the first to systematically evaluate prevention of both cervical and oropharyngeal cancer in a high-risk Indigenous population taking into account all population, testing, and surveillance options.

**Registered Report Identifier:**

RR1-10.2196/10503

## Introduction

### Human Papilloma Viruses

Human papillomaviruses (HPVs) are a heterogeneous group of over 100 genotypes, being circular, double-stranded DNA viruses that grow in stratified epithelia of skin and mucous membranes. There are approximately 15 HPV types that have potential to cause cancer. Before implementation of vaccination, a restricted number of these genotypes, known as high risk or oncogenic types, were the most common sexually transmitted infection in Australia, with an estimated 4 out of 5 Australians having a high-risk HPV (hrHPV) infection at some point in their lives [1]. The most common hrHPV types are HPV-16 and HPV-18. These HPVs are a precursor to a range of cancers in both females and males (particularly cervical cancer, other anogenital cancers and oropharyngeal cancer) and are usually acquired within 2-5 years of commencing sexual activity [2]. Preventing such HPV infections is a public health priority to reduce cancer and HPV-associated complications [3].

The rate of carriage of hrHPV at a population level at sites relevant to cancer (anogenital and oropharyngeal) in Australia is difficult to characterize; most surveys have not been national or representative, and almost all have focused on females only. Although there are some general population oral HPV DNA prevalence data [4], the big gap in the knowledge base is the oral and oropharyngeal HPV prevalence in a high-risk group for oropharyngeal cancers, Aboriginal and Torres Strait Islander Australians [5].

### Human Papillomavirus and Cervical Cancers

Cervical cancer is the fourth most common cancer of women internationally [6]. Virtually, all cervical cancers are attributable to infection with oncogenic genotypes of HPV [7]. In an international study, incidence of cervical cancer was found to be higher among Indigenous women than among non-Indigenous women in most countries (Australia, New Zealand, Canada, and the United States) [8]. In Australia, there are undisputedly higher rates of cervical cancer and mortality among Indigenous compared with non-Indigenous women [9].

### Human Papillomavirus and Oropharyngeal Cancers

Oropharyngeal cancers include cancer of the middle part of the throat: the tonsils, posterior one-third of the tongue, and lateral and posterior walls of the oropharynx [10]. Approximately 90% of oropharyngeal cancers are squamous cell carcinomas [11]. Tobacco and heavy alcohol use, often in a background of diets poor in essential antioxidant vitamins and minerals, are major risk factors [12]. The impact of tobacco and alcohol is synergistic, ie, a person exposed to both has multiplicative, not just additive, risk [13]. Additional risk indicators include being male, older age, having infection with *Candida* or a pro-inflammatory bacteriale, or a compromised immune system [14,15]. Survival from oropharyngeal cancers is comparatively low. This is because they are frequently asymptomatic and diagnosed at a late stage. In general, more than half of all persons with oropharyngeal cancer have regional or distant metastases at diagnosis [16]. Once the cancer has metastasized, prognosis is worse than when localized. Relative 5-year survival in the United States is 82% for localized disease, 56% for regional lymph node spread, and 33% for distant metastases [17].

In addition to tobacco and alcohol, HPV has been increasingly identified as a significant risk factor for oropharyngeal cancer [18]. Both oral HPV prevalence and HPV-positive oropharyngeal cancers are associated with younger age (compared with tobacco and alcohol-related oropharyngeal cancer), sex (higher incidence in males), sexual behaviors (higher among those who have ever had oral sex), and number of sexual partners (applies particularly to men, but works both ways) [19]. These factors increase the risk of cancer development 3-to 5-fold [20]. The proportion of oropharyngeal cancers that are HPV-positive has increased over the last decade in Europe and North America to an estimated 70% [21]. Indicative data from Australia suggest a similar increase in the fraction of oropharyngeal cancers that might be attributable to HPV [22].

### Burden of Oropharyngeal Cancer in Australia

Head and neck cancers (of which oropharyngeal cancer is one) have been described as being more emotionally traumatic than any other form of cancer [23,24]. Treatments can be debilitating and disfiguring, with patients frequently going on to live with chronic functional impairment in a range of areas including speech and swallowing [25]. There are substantial effects on oral health and nutrition [26], on social functioning, and on mood, with an often immediate decrease in health-related quality of life which persists long term [24]. Ariyawardana and Johnson reported that, although rates of overall lip, oral cavity, and oropharyngeal cancer declined between 1982 and 2008, presumably due to decreased alcohol and tobacco use, and potentially improved sun protection for lip cancer, they were still high [11]. When considered in isolation, rates of oropharyngeal cancer increased during this time (1.2% per annum for men, 0.8% per annum for females), possibly due to an increased incidence of HPV-related oropharyngeal cancer [11]. Hong reported that the proportion of oropharyngeal cancers which were positive for HPV DNA and p16 increased from 20.2% in 1987-1995 to 63.5% in 2006-2010 [27].

### Oropharyngeal Cancer in Aboriginal and Torres Strait Islander Australians

There is little documented evidence on the incidence of oropharyngeal cancer among Aboriginal and Torres Strait Islander Australians at a national level. Johnson and colleagues reported that Indigenous persons living in Queensland (who comprise 4% of the Queensland population) were more likely than the total Queensland population to be diagnosed with certain head and neck cancers between 1997 and 2012, specifically base of tongue or tonsil or oropharynx (standardized incidence ratio=2.16; n=81) [5]. Five-year cause-specific survival estimates, adjusted for age and sex, were 75% (95% CI 74-76%) for non-Indigenous persons and 43% (95% CI 38-49%) for Indigenous persons. Similar differentials in survival were observed for cancers of the base of tongue/tonsil/oropharynx (64% vs 27%) and mouth/oral cavity (66% vs 42%) [28]. In 2003, the rate ratio of disability-adjusted life years due to oral cavity and oropharyngeal cancer among Aboriginal and Torres Strait Islander Australians was 3.8 times that reported for the total Australian population [29].

### Oropharyngeal Cancer Risk Factors Among Indigenous Australians

As with the general population, alcohol and tobacco use are often cited to be significant risk factors for oropharyngeal cancer among Aboriginal and Torres Strait Islander Australians, although the population attributable fraction for HPV-related oropharyngeal cancers in this group is unknown [9]. Aboriginal and Torres Strait Islander Australians generally commence tobacco smoking at an earlier age, continue to smoke for longer, and make fewer quitting attempts than non-Indigenous Australians [30]. In 2012-2013, Aboriginal and Torres Strait Islander Australians were 2.6 times more likely than non-Indigenous Australians to be current daily smokers (40% compared with 15% after age standardization) [31].

### High-Risk Human Papillomavirus Among Aboriginal and Torres Strait Islander Australians

There are currently no population estimates of carriage of hrHPV in the upper aero-digestive tract among Aboriginal and Torres Strait Islander Australians. This is a substantial deficit in the contemporary knowledge base, particularly given the higher risk for oropharyngeal cancer among this population. To determine prevalence of hrHPV, and risk factors associated with the infection among this population, data on prevalence using sensitive HPV detection methods are necessary.

### Efficacy of Human Papillomavirus Vaccination

Prevention of acquiring a persistent infection with a hrHPV through vaccination is a cost-effective and life-saving intervention to decrease the burden of HPV-related cancers in Australia. Current bi- or trivalent vaccines are effective against the 2 genotypes most strongly associated with cancer (types 16 and 18), which are detected in approximately 95% of HPV-positive oropharyngeal tumors in the United States [32,33], 94% of HPV-positive oropharyngeal tumors in males in Australia [27], and approximately 70% of cervical cancer worldwide. HPV vaccination in Australia is currently provided free of charge to adolescents aged 12-13 years through a school-based program. The goal of early vaccination is to immunize before first exposure to hrHPV [34]. The efficacy and immunogenicity of hrHPV vaccines have proven excellent in several phase 2 and 3 trials involving tens of thousands of women [35]. Few subjects lost their antibodies during the 5-6 years after vaccination, with no breakthrough disease occurring among these individuals. There has been a move to a 2-dose vaccination, which means the effective vaccine coverage in the National HPV Vaccination Program Register in Australia is likely to go up (easier to deliver in 2 rather than 3 doses), although dose spacing is very important. The next generation of nonavalent vaccines [36] has also been approved and is currently under review. Importantly, there is now a growing body of evidence that current vaccines prevent HPV infection at noncervical sites, including the mouth and oropharynx [37,38].

### Human Papillomavirus Vaccination Uptake in Indigenous Populations

There are no national-level data available for the uptake of HPV vaccination among Aboriginal and Torres Strait Islander Australians. Data from the first stage of the National HPV Vaccination Program (NHVP) suggest that, in Queensland, coverage among Indigenous girls aged 12-17 years compared with all girls aged 12-17 years was lower with each dose (lower by 4% for dose 1, 10% percent for dose 2, and 15% for dose 3). This pattern was not seen in the Northern Territory, where initial coverage was 17% lower among Indigenous girls, but the course completion rate among those who started vaccination was identical (84%) [39]. Both used data on genital warts, and both reported that the impact of HPV vaccination appeared to be at least as strong in young Indigenous Australians as in non-Indigenous Australians [40,41]. Although there were initially catch-up phases of the NHVP that offered publicly funded vaccination to females aged up to age 26 years and boys aged up to 15 years, these ceased in 2009 and 2014, respectively. There is now a relatively narrow window in early adolescence when individuals can receive free vaccination; otherwise, the remainder or full vaccine course incurs an additional cost (approximately Aus $150 per dose). This is likely to be a substantial barrier to uptake among Aboriginal and Torres Strait Islander Australians, as is the possibility of culturally inappropriate, insensitive, alienating, or intimidating aspects of provision in broader health care services [42]. It is worth highlighting, however, that school-based vaccinations reduce disparities, with school retention rates among Indigenous preadolescents being reasonably high at age 12 to 13 years [43].

### Efficacy of Human Papillomavirus Vaccination in Older Populations

Wheeler and colleagues [44] conducted a phase 3, double-blind, randomized controlled trial among healthy women older than 25 years to test the hypothesis that the HPV 16/18 vaccine would be efficacious in protecting against infections, cytological abnormalities, and lesions associated with HPV 16/18 and cervical intraepithelial neoplasia level 1+, irrespective of HPV type, and infection with nonvaccine types HPV 31 and HPV 45. After 7 years of follow-up, their hypothesis was proved correct. HPV vaccination is available to females aged up to 45 years and males aged up to 26 years in Australia, at a cost. The level of elective uptake in females who were not eligible to receive vaccination through the publicly funded program is low (11%) [45].

### Cost-Effectiveness of Human Papillomavirus Vaccination

Although population-level impact and herd effects following HPV vaccination have been widely documented, cost-effectiveness evaluations in Australia are scarce. Kulasingam et al reported on the cost-effectiveness for females, which supported the original Commonwealth Serum Laboratories application for the vaccine to be included on the National Immunization Program [46]. The cost-effectiveness evaluations that supported HPV vaccination for boys have never been published, although Smith and colleagues [47] evaluated the herd immunity benefits and incremental impact of male vaccinations on cancer, whereas Simms et al [48] evaluated whether cervical screening would remain cost-effective in women offered the next generation nonavalent HPV vaccine in 4 developed countries. To the best of our knowledge, there have been no specific cost-effectiveness evaluations of the optimal strategies for HPV vaccination in Indigenous populations (including the potential for extending vaccination to older ages)—a critical gap in the knowledge base given the higher burden of oropharyngeal and cervical cancer risk among this group.

### What Are Utilities and Why Are They Important?

Utilities are fundamental values that represent the strength of an individual’s preferences for specific health-related outcomes. Measuring health utilities involves 2 main steps: defining a set of health states of interest and valuing those health states. It is important to estimate utilities in relation to HPV, cervical cancer, and oropharyngeal cancer among Aboriginal and Torres Strait Islander Australians because the frame of reference regarding prevention, screening, and burden of cancer treatment is likely to differ in meaningful ways compared with the non-Indigenous population. Differences may be because of the substantial travel required for many Indigenous Australians and because of time away from family and country. There may be inherent distrust and fear of hospital systems not apparent in non-Indigenous populations, and the specific treatment-associated morbidity may be valued differently. It is important to capture this information that can be used to directly calculate quality-adjusted life years and to, in turn, be translated into health policy regarding Aboriginal patient journeys with primary and secondary prevention for cervical, other genital, and oropharyngeal cancer. Although health state valuations appropriate for modeled economic evaluations have been undertaken for cervical HPV disease including cancer and precancerous lesions [49,50] and for genital warts [51], there is a paucity of information on health state valuations for other HPV cancer states including oropharyngeal cancer. There is a particular dearth of information on HPV-related health state valuations for Aboriginal and Torres Strait Islander Australians.

### Study Aims

The aims of this study were as follows:

1. To yield population estimates of the age-specific prevalence of oncogenic genotypes of HPV in the mouth and oropharynx of defined Aboriginal and Torres Strait Islander populations (male and female). *Hypothesis: The prevalence of oral HPV among Aboriginal and Torres Strait Islander Australians will be high compared with national-level estimates.*
2. Using preliminary data from Aim 1, and information on the prevalence of other risk factors for oropharyngeal cancer in the Aboriginal and Torres Strait Islander population, to estimate burden of HPV-related oropharyngeal cancer among Aboriginal and Torres Strait Islander men and women. *Hypothesis: The burden among Aboriginal and Torres Strait Islanders of HPV and related oropharyngeal cancer will be high.*
3. To estimate the impact of HPV vaccination as currently implemented on rates of cervical and oropharyngeal cancer among Aboriginal and Torres Strait Islander Australians. *Hypothesis: HPV vaccination will, over time, reduce the burden of cervical and oropharyngeal* *cancer among Aboriginals and Torres Strait Islanders.*
4. To evaluate efficacy and cost-effectiveness of targeted extended HPV vaccination among Aboriginal and Torres Strait Islander Australians, incorporating the effectiveness against both cervical cancer (in females) and oropharyngeal cancer. Different upper age thresholds for targeted extension will be considered. *Hypothesis: Age-extended HPV vaccination for Aboriginal and Torres Strait Islander Australians will be efficacious; we will estimate an upper age limit at which it would be cost-effective.*

## Methods

### Study Design

Our overall study design will impose minimal impost on Aboriginal and Torres Strait Islander participants. It involves testing for carriage of hrHPV in the mouth and oropharynx among 1000 Aboriginal South Australians by simple saliva collection, with follow-up at 12 and 24 months, collection of sexual history at baseline, collection of information for estimating utilities at baseline, genotyping the viruses, statistical analysis (including cost-effectiveness modeling), data interpretation and development of vaccination and clinical therapeutic strategies to better communicate the benefits of HPV vaccination and lifestyle changes in cancer prevention, and approaches to take into account the preferences of Indigenous people in prevention and management of cancer driven by the Aboriginal community.

### Ethical Approval

Ethics approval has been obtained from the University of Adelaide Human Research Ethics Committee (H-2016-246). Before being recruited, all participants will be required to sign an informed consent form, which includes consent for the authors to publish the findings in the peer-reviewed scientific literature. The authors confirm that supporting data and material in the study will be made available through Springer Nature’s Data Support Services.

### Study Population and Recruitment

We will recruit 1000 Aboriginal South Australian male and female adults, with a focus on Port Augusta, Whyalla, Port Lincoln, Mount Gambier, Ceduna, and Adelaide. Census data indicate approximately 22,000 Aboriginal adults reside in these areas. The investigators have a 13-year relationship with key Aboriginal stakeholder groups in these locations, who are willing and excited to be part of the study. Recruitment strategies will be based on those successfully implemented in the past, including the following: establishing service agreements with key Aboriginal community-controlled health organizations, liaising with community champions previously involved in our research, and encouraging word-of-mouth spread of knowledge.

### Inclusion and Exclusion Criteria

Participants will be aged 18+ years, identify as being Aboriginal or Torres Strait Islander, and planning to live in South Australia for the next 3 years. Participants not enrolled during the original recruitment period will not be eligible to participate in the follow-up phases.

### Collection of Human Papillomavirus and Oropharyngeal-Related Information

Permission to obtain sensitive information from participants will be sought, with relevant information related to alcohol and tobacco use, HPV diagnosis, health behaviors (including HPV vaccination status and sexual behaviors), and social determinants asked through a self-report questionnaire. Data will be collected by experienced Aboriginal research officers.

### Collection of Utilities Information

We will design a questionnaire for the utility study, in which all 1000 participants taking part in the oral HPV prevalence study will be asked to indicate preferences (rank and utility scores) for 6 hypothetical states relating to oral HPV testing, precursor oropharyngeal cancer, and early-stage oropharyngeal cancer (including examinations and treatment). On the basis of standard methods used in the development of utilities, preferences for health states will be measured through ranking (1 through to 6), followed by a 2-stage standard gamble. We will focus on valuing the long-term oropharyngeal cancer health state of the average patient who survives up to 5 years after diagnosis and treatment, which is the most appropriate state for modeling cost-effectiveness of prophylactic HPV vaccination. We will seek to generate utility values for all cancer stages at diagnosis. The process for developing the health states will involve the following: (1) the most common stage(s) of HPV-associated oropharyngeal cancer at diagnosis identified from the literature; (2) the recommended treatment for the relevant stages(s) of oropharyngeal cancer identified and confirmed from published studies; and (3) the more common long-term consequences (applying to ≥50% patients) in patients surviving the initial treatment phase described based on the literature, and subsequent refinement by clinical experts involved in managing oropharyngeal cancer [52,53].

### Collection of Oral Human Papillomavirus Data

All participants will be asked to provide a saliva sample using a commercially available kit (Omnigene 501; DNA Genotek Inc, Canada) from which microbial DNA for genotyping will be extracted. This involves the participant: (a) not eating or drinking for 30 min before collection; (b) spitting until 2 ml reaches the fill line on the container (takes 2-3 min); (c) closing the lid on the funnel (to release preservative liquid into tube); (d) removing the funnel lid on the container; and (e) placing the small cap on the tube and shaking the tube for 5 seconds. This results in over 100 μg of DNA collection, which is sufficient for the testing required. The sample can be kept at room temperature (for up to 12 months) until collection by the Aboriginal research assistants, who will send it to an appropriate laboratory for analysis. Saliva samples will be collected at baseline, 12 months, and 24 months.

### Data Analysis

In brief, the analysis plan for each aim is described below.

#### Aim 1: To Yield Population Estimates of Oral Human Papillomavirus in the Aboriginal and Torres Strait Islander Population

##### DNA Extraction and Quality Check

Antonsson and colleagues have evaluated 3 different kits (all semi-automated) for DNA extraction, namely, Promega’s Maxwell-16 Viral Total Nucleic Acid Purification Kit, QIAGEN’s QIAamp Mini Elute Virus Spin Kit, and QIAamp Blood DNA Mini Kit (QIAcube). We will use the Promega Maxwell viral kit for DNA extraction as the DNA yield and quality was superior compared with the 2 other kits. β-globin polymerase chain reaction (PCR) with the primers PCO3 and PCO4 will be carried out on all samples to ensure that they contain enough cells to detect human DNA, and that no PCR inhibiting agents are present [54].

##### HPV Type Determination

We will analyze all samples with the optimized general primer (GP)+PCR system that detects most mucosal HPV types and all hrHPV types that have oncogenic potential in mucosal tissue [54]. All HPV DNA positive samples will be sequenced to confirm viral DNA sequences. For the sequencing, HPV-positive PCR products will be purified with the Agencourt AMPure PCR purification kit in a magnetic 96-ring SPRIplate. Sequencing reactions containing the purified PCR products together with GP+primer and BigDye Terminator will be performed. Sequence reactions will be purified with the Agencourt CleanSEQ dye-terminator removal kit in a magnetic 96-ring SPRIplate. Direct sequencing will be carried out initially. Samples with multiple HPV types will be cloned before sequencing, with at least 5 clones sequenced per sample. Sequence reactions will be analyzed with an automated DNA sequencer (ABI model 3100). The DNA sequences will be compared with available sequences in GenBank through the BLAST server. We have chosen a standard PCR method, which has been used in several projects by Antonsson and proven to be both reliable and reproducible [4,55-57].

##### Specimen Variables

HPV status and genotypes found will be analyzed. The genotypes will also be divided into low-risk (not found in cancer; eg, HPV-6 and -11) and hrHPV types (found in cancer; eg, HPV-16 and -18). As multiple HPV infections are likely, incident infection will be defined as a new type-specific HPV infection not detected in a previous sample. We will determine the precision of HPV prevalence estimates obtainable with the sample of 1000 in age- and sex-specific subgroups. We will base these estimates on measurements in other populations by age and sex, and then characterize 95% CIs obtainable in the given sample size.

#### Aim 2: Using Preliminary Data From Aim 1, and Information on Prevalence of Other Risk Factors, to Estimate Burden of Human Papillomavirus-Related Oropharyngeal Cancer Among Aboriginal and Torres Strait Islander Australians

Sufficiently detailed information on oropharyngeal cancer rates overall, or the proportion of oropharyngeal cancers which are HPV-positive, are not available for the Indigenous population. Therefore, these rates will be estimated using published data on HPV-positive and HPV-negative oropharyngeal cancers in the general population [27,58]. Each estimate will be scaled to account for different risk factor prevalences. For HPV-positive cancers, we will use our preliminary findings on the relative prevalence of oral HPV in the Indigenous population compared with that in the general population [57] to scale rates of HPV-positive oropharyngeal cancer. Overall estimates of oropharyngeal cancer will be compared with available published data on the relative incidence of oropharyngeal cancer overall in the Indigenous versus the general population to ensure consistency [5].

##### Utilities and Costings

We will perform systematic reviews of utilities for oropharyngeal cancer diagnosis, surveillance, surgery, and the diagnosis/treatment of associated cancers. We will also perform systematic reviews of complication rates and associated utilities for surveillance and surgery. Aggregate costs for each step involved in screening, diagnosis, family counseling, referral and management pathways, and cancer diagnosis and treatment will be collated using methods previously employed by Canfell and colleagues for other cancer-related applications [59,60]. Briefly, detailed clinical pathways for current practice will be described using relevant patterns of care studies and clinical practice guidelines, and will take into account different patterns of care/attendance for treatment among Aboriginal and Torres Strait Islander Australians. Item costs of the component services will be obtained from the Medicare Benefit Schedule Online for outpatient medical services, the latest available National Hospital Cost Data Collection Round for inpatient services and the Pharmaceutical Benefits Schedule Online.

#### Aim 3: To Evaluate the Impact of Human Papillomavirus Vaccination as Currently Implemented on Oropharyngeal and Cervical Cancer Rates Among Aboriginal and Torres Strait Islander Australians

The results of Aim 2 will feed into an existing model of HPV transmission, vaccination, and natural history developed by Canfell and colleagues through previous grants from Australia’s National Health and Medical Research Council (NHMRC). This model has been used extensively for evaluations around HPV and cervical cancer prevention in government-commissioned reports and in 20 journal publications. In the proposed study, this model will be further tailored to the Aboriginal and Torres Strait Islander population. The developed model will be used to make detailed predictions of the impact of HPV vaccination over time on oropharyngeal and cervical cancer among Aboriginal and Torres Strait Islander Australians, including under a range of age ranges for vaccination and dose/uptake assumptions. This will be based on the burden of HPV-attributable oropharyngeal and cervical cancer in the Indigenous population estimated in Aim 2, which will take into account different tobacco smoking prevalence or varied attributable fraction to allow for the higher proportion of HPV-negative tumors in the Indigenous population. Estimates of HPV vaccine uptake in Aboriginal and Torres Strait Islander Australians will be used, in conjunction with available data on HPV vaccine impact in Aboriginal and Torres Strait Islander populations or precursor/proxy outcomes, such as prevalence of infection with vaccine-included types and anogenital warts. We will validate model predictions against these previously reported outcomes [41,42].

#### Aim 4: To Evaluate Efficacy and Cost-Effectiveness of Targeted Extended Human Papillomavirus Vaccination on Oropharyngeal Cancer Among Aboriginal and Torres Strait Islanders, Incorporating the Effectiveness Against Both Cervical Cancer (in Females) and Oropharyngeal Cancer

We will use data from the literature review and utility estimations in Aim 2 to inform model assumptions on the demographics and risk profile of the population. To achieve higher coverage in this group, we will estimate impact and cost-effectiveness of funding extended catch-up vaccination for Aboriginal and Torres Strait Islander Australians (for those who did not receive the vaccine through the school-based program). A range of potential extended catch-up strategies will be considered, eg, funding HPV vaccination for females ± males aged up to 18, 25, 30, or 45 years who have not already received a full course. We will consider both first and second generation HPV vaccines and also consider strategies involving revaccination of individuals who have already received the first generation vaccine with the second generation vaccine. We will also consider different potential delivery mechanisms, eg, via Aboriginal health services and/or other community providers. For each analysis, a large “virtual” sample of the Aboriginal and Torres Strait Islander population will be simulated. Each evaluation will then use all fitted parameter sets to derive a baseline result and 95% CI. These evaluations will simulate 10,000 individuals. All evaluations will be accompanied by extensive sensitivity analysis, using one-way and probabilistic sensitivity analysis techniques that will take into account the full range of identified fitted parameter sets. For each strategy, we will calculate a number of measures of effectiveness, including change in cancer incidence, mortality, life years saved, and quality-adjusted life years gained. We will also estimate absolute case numbers based on population projections available by age, sex, and calendar year for ASTI Australians. We will assess morbidity via calculation of quality-adjusted life years and calculate complication numbers from surveillance and surgery. We will take into account varying rates of attendance for treatment in the Indigenous population [61], but consider a range of assumptions in sensitivity analysis. We will provide detailed predictions of health resources utilization, including numbers of tests, biopsies, and treatments. We will assess costs of diagnosis, surveillance and cancer treatment, and the total budget impact for each year from 2017 to 2030. We will calculate incremental cost-effectiveness ratios for both life years saved and quality-adjusted life years. If results suggest that extended HPV vaccination among Aboriginal and Torres Strait Islander Australians is not cost-effective at any age threshold, we will perform threshold analysis to determine the cost at which extended HPV vaccination to different upper age thresholds becomes cost-effective.

### Ethical Approval

Ethical approval for this study has been obtained by the University of Adelaide Human Research Ethics Committee (H-2016-246).

## Results

Participant recruitment for this study commenced in February 2018 and enrollment is ongoing. The first results are expected to be submitted for publication in 2019.

## Discussion

### Study Overview

Oropharyngeal cancer is an important cancer affecting Aboriginal and Torres Strait Islander Australians at a higher rate than other Australians. Infection with hrHPV is a significant risk factor for oropharyngeal cancer. HPV vaccination is effective against the 2 most common types of hrHPV, with some promise that current vaccines may prevent oral infections (potentially reducing the risk of oropharyngeal cancer). Given the elevated risk of HPV-related cancers in this group, it may be reasonable to extend the comparatively brief timeframe (when aged 12-13 years) in which Aboriginal and Torres Strait Islander Australians can access an otherwise costly vaccine via public funding. The project will have a number of important outcomes. Synthesis of evidence will directly support estimates of the burden of oropharyngeal cancer among Aboriginal and Torres Strait Islander Australians and the effectiveness and cost-effectiveness of prevention. This will be important for health services planning, and for Aboriginal health worker and patient education. The project provides a key example of how carefully calibrated, data-driven disease models can integrate with Aboriginal community views and expectations to estimate disease burden and to guide policy decisions.

### Study Strengths

The strengths of the study include it being the first to obtain and link all the information on cervical and oropharyngeal cancers via modeling (possibly in any population but certainly for the Australian Aboriginal and Torres Strait Islander population) and the focus on engagement, enabling community and individual preferences to play a large role in decision making for Aboriginal and Torres Strait Islander Australians. Burger and colleagues investigated the impact of HPV on 6 HPV-associated cancers, including cervical and oropharyngeal cancer, among 5 ethnic groups in the United States, one of which included Native American/Alaskan Natives [62].

### Study Limitations

The limitations include the sample frame for oral HPV prevalence assessment being pragmatic, ie, utilizing convenience sampling methodology rather than attempting to be representative. Although there is a risk of sampling bias, the efforts required to obtain a representative sample are recognized as being both expensive and time-consuming [63]. Additionally, it is recognized that oral HPV measurement through saliva sampling is blunt as it does not provide a direct measure of HPV exposure at each potential cancer site. Finally, as in any modeled assessment, assumptions about future vaccination and screening coverage need to be made, but we remain committed to engaging the Aboriginal and Torres Strait Islander community in consultation about such assumptions via the study’s Aboriginal Reference Group.

## Appendix

Multimedia Appendix 1 Peer Review Assessment from Australia's National Health & Medical Research Council

## Abbreviations

GP: general primers
HPV: human papillomavirus
hrHPV: high-risk human papillomavirus
NHMRC: National Health and Medical Research Council
NHVP: National HPV Vaccination Program
PCO3: (5’CTTCTGACACAACTGTGTTCACTAGC3’) oligonucleotide
PCO4: (5’TCACCACAACTTCATCCACGTTCACC3’) oligonucleotide
PCR: polymerase chain reaction
