# Supplementary material for: Human Papillomavirus and Oropharyngeal Cancer Among Indigenous Australians: Protocol for a Prevalence Study of Oral-Related Human Papillomavirus and Cost-Effectiveness of Prevention
Source: JMIR Res Protoc. 2018 Jun 8;7(6):e10503. doi: 10.2196/10503 (PMC6015268; doi:10.2196/10503)
Supplement: Multimedia Appendix 1 [file resprot_v7i6e10503_app1.pdf]

Australian Government
National Health and Medical Research Council

NHMRC

GPO Box 1421 | Canberra ACT 2601
16 Marcus Clarke Street, Canberra City ACT 2600
T. 13 000 NHMRC (13 000 64672) or +61 2 6217 9000
F. +61 2 6217 9100
E. nhmrc@nhmrc.gov.au
ABN 88 601 010 284
www.nhmrc.gov.au

Associate Professor Lisa Jamieson
lisa.jamieson@adelaide.edu.au

Dear Associate Professor Jamieson

Application ID: **APP1120215**
Type: **Project**
Application Title: **HPV and oropharyngeal cancer in Indigenous Australians**

Thank you for applying for National Health and Medical Research Council (NHMRC) Project funding commencing in 2017.

You are now invited to respond to assessor comments by submitting your Applicant Response (rebuttal). Assessor comments for your application are at Attachment A.

Your rebuttal must be uploaded into RGMS by **23:59 hrs AEST/AEDT Monday, 27 June 2016** to ensure that it is provided to the relevant Peer Review Panel for consideration along with your application. Rebuttals received after this time will not be accepted. Instructions for submitting your rebuttal are provided in the RGMS User Guide – Assessment Processes.

Please ensure your rebuttal is a single PDF document that meets the size, length and formatting requirements outlined at Attachment B. Rebuttals that do not adhere to these formatting requirements will be excluded from consideration.

As your response is limited in length, please use your judgement in responding to assessors' comments and address the most important issues. The rebuttal should be used to clarify queries raised by the reviewers and not to propose changes to the project plan, methodology or team membership.

If you consider that your assessor report contains biased or inappropriate comments, you should contact your Research Administration Officer (RAO). RAOs may then contact projects@nhmrc.gov.au to raise your concerns and to seek the NHMRC's advice on how they may be addressed.

It is important to raise potential concerns regarding the appropriateness of assessor comments during the applicant response period. Complaints received after peer review will be difficult to address.

Applicants should continue to address other elements of the Assessor's Report while NHMRC is reviewing your concerns. NHMRC will notify the RAO of the review's outcome.

If you have questions regarding the rebuttal process, please contact NHMRC's Research Help Centre at help@nhmrc.gov.au or on 1800 500 983.

Yours sincerely,

Research Programs

Encl.

Attachment A: Assessor report
Attachment B: Applicant Response formatting requirements

Australian Government
National Health and Medical Research Council

NHMRC

GPO Box 1421 | Canberra ACT 2601
16 Marcus Clarke Street, Canberra City ACT 2600
T. 13 000 NHMRC (13 000 64672) or +61 2 6217 9000
F. +61 2 6217 9100
E. nhmrc@nhmrc.gov.au
ABN 88 601 010 284
www.nhmrc.gov.au

Attachment A: Project 2016 Assessor Report

**Application ID**	APP1120215
**CIA Name**	Associate Professor Lisa Jamieson
**Application Title**	HPV and oropharyngeal cancer in Indigenous Australians

Spokesperson
01. Scientific Quality

Meets score for criteria

Spokesperson
02. Significance of the expected outcomes AND/OR Innovation of the concept

Meets score for criteria

Spokesperson
03. Team quality and capability – relative to opportunity

Meets score for criteria

Spokesperson
Budget Comments

None provided.

Spokesperson
Overall Comments

None provided.

__External Assessor__
01. Scientific Quality

This is an excellent, original proposal with clearly specified hypotheses. Strengths include the outstanding experience of the research team, strong Aboriginal representation and world-leading, proven approach to cost-effectiveness modelling. The measurement of prevalence is methodologically straightforward although logistically will be difficult in remote regions with consideration of cultural issues. The analysis of prevalence, vaccine impact and efficacy and cost-effectiveness involves proven statistical models for evaluating vaccine impact in Australia and internationally by CI Canfell. Laboratory analys is being conducted using appropriate methods, although the laboratory facilities where the work is being undertaken are not described. Specific questions: Sample size calculations are not presented. Is 1000 sufficient for stratification by gender and age specific sub-groups at the expected prevalence?

- The proposal states that the researchers perceive no difficulties in recruiting 1000 adults and had recruited amongst 500 within 2 weeks over a 2 week period for another project. This raises the question as to why the recruitment is planned over such a long time – it appears to be 18 months. It should be possible to complete baseline recruitment within 6 months given past experience and then allow up to 24 months for follow up. However, the investigators have not explained the requirement for 24 months of follow up of all participants, not just those HPV+ This is not described specifically within Aim 1 yet will be a major cost component. Please provide further detail about the number of people to be followed for 12 and 24 months and how this information will be used in the modelling.
- How will HPV+ people be managed clinically?
- Is the collection of utilities information culturally specific and account for literacy/ languages? Probabilities for gambling can be an abstract concept – will this be tested in this population prior to the field?

__External Assessor__
02. Significance of the expected outcomes AND/OR Innovation of the concept

This project addresses a previously understudied issue and has the potential to significantly contribute to the health of indigenous Australians. The HPV vaccine has already had a major impact in Australia and could be further used to close the cancer gap specifically for indigenous Australians. This project builds upon experience and modelling already developed by the research team through a new collaboration and is also likely to be relevant to indigenous populations in other countries. I believe the significance of this grant is outstanding.

__External Assessor__
03. Team quality and capability – relative to opportunity

This is an outstanding team of researchers who are extremely well qualified to deliver the outcomes of this project. CIA has an international reputation and track record in Indigenous Oral health and has worked with most of the other investigators. The collaboration between CIA's group and CIB's group appears to be new and builds on the strengths and experience of both teams. The team includes many indigenous investigators and an Aboriginal Reference Group and CIA Jamieson's team have longstanding experience in indigenous health.
My one question relates to CIA's available time commitment to the study. As an outstanding researcher she has delivered strongly on 2 previous NHMRC grants as CIA and has recently been awarded 4 grants (3 commenced in 2015 and 1 in 2017) as CIA totalling almost $4 mill. The early years of co-ordinating projects are intense and this project will be highly dependent on the CIA to provide oversight and high level co-ordination. A project manager will be appointed but this will be a new appointment and this person will need to come up to speed. What will be the CIA's time commitment to this grant and how will this fit with the other NHMRC funded grants?

External Assessor
Budget Comments

The budget for this grant is significant, and the major costs are salaries and travel to recruit and follow up 1000 people. As per my question under scientific quality, further detail is required on the timing of baseline recruitment and co-ordination of follow up and vaccination. Given past experience of the research team, baseline recruitment could be completed within 6 months. It would be helpful to see a more detailed schedule of proposed numbers to be recruited along with vaccine and follow up visits by year– is it possible to reduce the duration of recruitment and therefore the study period?
I strongly support the aim to offer vaccine to all participants, even though this is not an intervention. However, the vaccination schedule does not align with the recruitment and follow up of participants. One research nurse has been assigned to deliver the vaccine and will have to do this at every baseline visit and at 1 and 6 months. Further follow up samples are then collected at 12 and 24 months. This is logistically a difficult task in remote areas. Please clarify the total amount of budget for HPV vaccination (including salaries and travel). Could any funding be obtained from philanthropic sources for the vaccination, as unfortunately the timing of vaccine doses does not align with follow up visits at 12 and 24 mths? DNA extraction is specified for 1000 samples and then 500 in year 1 and 4. 3000 samples are referred to for HPV type determination, saliva and also reimbursement. Why are all 1000 participants being followed up twice ie even HPV negative participants? Please see comment under Scientific quality and provide further detail on this aim.

External Assessor
Overall Comments

As included under specific criteria above.

External Assessor
01. Scientific Quality

This study will add data on prevalence and risk factors for oral HPV infection among Aboriginal and Torres Strait Islander Australians. This data is currently lacking from the evidence base. The study team have strong Aboriginal representation and have demonstrated considerable engagement with, and support from key stakeholder to conduct the study. Given this, the proposed recruitment targets of 1000 participants is highly feasibly.
However, the research plan lacks clarity. In particular, given the main outcome of this study is prevalence, it is not clear why additional oral samples are being collected and tested for HPV at 12 and 24 months? Are the investigators looking at oral HPV natural history? If so, how will this data be used to address the aims of the proposal? If not, why are samples being collected longitudinally? Could the timeline for the project and the budget be significantly reduced?
Further to this, the investigators propose to offer a 3-dose course of the quadrivalent vaccine to 30–45 year old, presumably female participants, starting at the baseline visit. How will this impact on the oral HPV measurements at follow-up? The bivalent vaccine has been shown to be efficacious against oral infections (Beachler et al. JNCI 2016) – efficacy of the quadrivalent vaccine is likely to be non-inferior.
Will male participants be offered vaccination?
Can the investigators please justify the chosen sample size of 1000 participants? What ratio of males to females will be recruited?
Can the investigators elaborate on the proposed genetic analysis as outlined in the research plan?

External Assessor
02. Significance of the expected outcomes AND/OR Innovation of the concept

The team proposes to use readily available data to model the cost-effectiveness of extending HPV vaccination for Aboriginal and Torres Strait Islander women against cervical cancer. This is a very valid undertaking with significant policy implications given the high burden of cervical cancer in this population. In fact there are currently a number of initiatives underway internationally aimed at extending HPV vaccination in adult women up to 45 years, particularly among female populations with high incidence rates of cervical cancer and low screening (HPV-FASTER; Bosch et a. Nature Review 2015).
How much of this work can therefore be done first, without the need for generating additional data on oral HPV prevalence? If extending female vaccination proves to be cost-effective against cervical cancer without considering oropharyngeal cancers, wouldn't this in itself provide strong evidence in favour of this strategy?
Further to this, though routine vaccination of males is recommended as part of Australia's vaccination schedule, cost-benefit analyses has already shown that increasing vaccine coverage in females would be more effective in reducing HPV infection and HPV related disease in males, through herd immunity.
Also, if extending catch-up strategies is proven to be cost effective with the inclusion of males, what further steps would be required to translate this into policy given the vaccine is only licensed in males up to the age of 26 years.
Lastly, given the very high rates of alcohol consumption and smoking in Aboriginal and Torres Strait Islander men, can the authors comment on what proportion of oropharyngeal cancers are likely to be attributed to HPV infection?

External Assessor
03. Team quality and capability – relative to opportunity

The project team have an outstanding track record in this area, ranging from very good to excellent. Importantly the research team comprises a very good mix of expertise appropriate to the proposal. There is no reason to question their commitment to this area of study nor their capacity to complete the study as planned.

External Assessor
Budget Comments

Given the project focuses on collecting prevalence data, further justification is needed to account for the allocation of funds for sample collection and testing at the follow-up visits.
Have the investigators factored in the cost of providing the quadrivalent vaccine?

External Assessor
Overall Comments

None provided.

Indigenous criteria comments (if applicable)

Spokesperson
Q01. Indigenous Criteria - Community Engagement

Meets score for criteria

Spokesperson
Q02. Indigenous Criteria - Benefit

Meets score for criteria

Spokesperson
Q03. Indigenous Criteria – Sustainability and Transferability

Meets score for criteria

Spokesperson
Q04. Indigenous Criteria – Building Capability

Meets score for criteria

Attachment B: Applicant Response formatting requirements

**Component**	**Requirements**
Format	A single document converted into a PDF file that must not exceed 2Mb in size.<br>Applicants and RAOs are advised to retain a copy of the response, including a copy of the PDF file they submit.
Page Limit	Not more than 2 pages. References and updates to the Chief Investigator's publication list must be included within the page limit.<br>Note: Applications that addressed the *Indigenous Research Excellence Criteria* (refer to http://www.nhmrc.gov.au/book/nhmrc-funding-rules-2015/6-assessment-criteria) will be permitted to use **an additional (third) page** to respond to the feedback provided for this component of their research proposal**.**
Paper Size	Standard A4 (210 x 297mm).
Header	The header is allowed outside the margin rules but must be at least 1cm from the top of the page.<br>The header must include the title "Applicant Response" in centred font with the Application ID in the top, right corner.
Margins	All margins must be at least 2cm.
Font	At least 12 point and Times New Roman only.
Line Spacing	Must be set to single or greater.
Character Spacing	Spacing must be set to normal. Scale must be set to 100%
Web Links	Do not include links to additional information on any website in the Applicant Response.
Graphics	Graphics (pictures, diagrams etc.) may be included in the response. Please note that the Applicant Response may be printed in black and white and any colour graphics must be visible and labelled appropriately.
Tables	Tabulated information containing text is not considered to be an image or diagram.<br>Text within tables must comply with the above requirements concerning fonts and spacing.
Labelling Graphs and Images	Axes of graphs and labels of parts of images must be no less than 10 Point Times New Roman font. The description and/or legends of all graphs and images must comply with the above formatting requirements.
File name	PDF file must be named in the following format: "Application ID - CIA Surname - Applicant Response.pdf" (for example: APP1098765 - Smith - Applicant Response.pdf).
